# Inflammatory Monocytes Orchestrate Innate Antifungal Immunity in the Lung

**DOI:** 10.1371/journal.ppat.1003940

**Published:** 2014-02-20

**Authors:** Vanessa Espinosa, Anupam Jhingran, Orchi Dutta, Shinji Kasahara, Robert Donnelly, Peicheng Du, Jeffrey Rosenfeld, Ingrid Leiner, Chiann-Chyi Chen, Yacov Ron, Tobias M. Hohl, Amariliz Rivera

**Affiliations:** 1 Rutgers, New Jersey Medical School, Department of Pediatrics, Center for Immunity and Inflammation, Newark, New Jersey, United States of America; 2 Rutgers, Graduate School of Biomedical Sciences, Newark, New Jersey, United States of America; 3 Fred Hutchinson Cancer Research Center, Vaccine and Infectious Disease Division, Seattle, Washington, United States of America; 4 Rutgers, New Jersey Medical School, Molecular Resource Facility and High Performance and Research Computing Group, Office of Information Technology, Rutgers University, Newark, New Jersey, United States of America; 5 Memorial Sloan Kettering Cancer Center, Sloan Kettering Institute, New York, New York, United States of America; 6 Rutgers, Robert Wood Johnson Medical School, Department of Pharmacology, Piscataway, New Jersey, United States of America; McGill University, Canada

## Abstract

*Aspergillus fumigatus* is an environmental fungus that causes invasive aspergillosis (IA) in immunocompromised patients. Although -CC-chemokine receptor-2 (CCR2) and Ly6C-expressing inflammatory monocytes (CCR2^+^Mo) and their derivatives initiate adaptive pulmonary immune responses, their role in coordinating innate immune responses in the lung remain poorly defined. Using conditional and antibody-mediated cell ablation strategies, we found that CCR2^+^Mo and monocyte-derived dendritic cells (Mo-DCs) are essential for innate defense against inhaled conidia. By harnessing fluorescent Aspergillus reporter (FLARE) conidia that report fungal cell association and viability in vivo, we identify two mechanisms by which CCR2^+^Mo and Mo-DCs exert innate antifungal activity. First, CCR2^+^Mo and Mo-DCs condition the lung inflammatory milieu to augment neutrophil conidiacidal activity. Second, conidial uptake by CCR2^+^Mo temporally coincided with their differentiation into Mo-DCs, a process that resulted in direct conidial killing. Our findings illustrate both indirect and direct functions for CCR2^+^Mo and their derivatives in innate antifungal immunity in the lung.

## Author Summary

Despite the significant impact of fungal infections to human health our understanding of immunity to these pathogens remains incomplete. Human mycoses are associated with high morbidity and mortality, even with modern antifungal therapies. *Aspergillus fumigatus* is the most common etiologic agent of invasive aspergillosis (IA), a serious infection that develops in immunodeficient patients. In this study we employ a combination of cell ablation strategies to examine the role of CCR2^+^Ly6C^+^ inflammatory monocytes (CCR2^+^Mo) in innate responses against a pulmonary infection with *A.fumigatus* conidia. We find that CCR2^+^Mo and their derivative dendritic cells (Mo-DCs) are required for defense against IA and that mice lacking these cells succumb to infection with *A.fumigatus*. Our studies indicate that CCR2^+^Mo and Mo-DCs exert crucial innate antifungal defense by two main mechanisms: 1) CCR2^+^Mo and Mo-DCs are a significant source of inflammatory mediators that augment the killing capacity of neutrophils and 2) conidial uptake by CCR2^+^Mo is coincident with their differentiation into Mo-DCs that directly kill fungal conidia via partially NADPH oxidase-dependent mechanisms. In aggregate, our studies find a novel essential function for CCR2^+^Mo in innate defense against a pulmonary fungal pathogen by mediating indirect and direct containment of fungal cells at the portal of infection.

## Introduction

The incidence of fungal infections has been on the rise for several decades due to increased use of immunosuppressive and myeloablative therapies for malignant and non-malignant diseases [1], [2], [3]. Invasive aspergillosis (IA), most commonly caused by *A. fumigatus*, is a frequent cause of infectious morbidity and mortality in patients with leukemia and in allogeneic hematopoietic cell transplant (HCT) recipients [4], [5], [6], [7].

Previous studies have determined that innate and adaptive components of the immune system play essential roles in defense against IA [2], [4], [8], [9], [10], [11], [12], [13], [14], [15], [16], [17]. Neutrophils have long been recognized as an essential innate cell in defense against IA, as neutropenia represents an important clinical risk factor [18]. Human susceptibility to IA in patients with defective neutrophil function (e.g. patients with chronic granulomatous disease) underscores the functional role of neutrophils in host defense. These findings are recapitulated in animal models of IA in which antibody-mediated depletion of neutrophils leads to uncontrolled fungal growth in the lung and to mortality from IA [19], [20], [21], [22], [23]. In addition to neutrophils, protective immune functions have been ascribed to a variety of innate cells that include macrophages, NK cells, myeloid DCs and plasmacytoid DCs [17], [19], [21], [24], [25]. While alveolar macrophages are capable of conidial killing in vitro [26] and in vivo [27], and likely contribute to innate defense, clodronate-mediated alveolar macrophage ablation did not lead to IA, suggesting that AM fungicidal activity can be functionally compensated by other leukocytes in vivo [21]. Similarly, the contributions of NK cells and myeloid DCs to antifungal defense against aspergillosis have been examined only in neutropenic mouse models of IA [24], [25]. Thus, despite the important contributions of other innate cells subsets in antifungal immunity, previous studies suggest that neutrophils are the sole indispensable innate effector cell in host defense against IA [19], [20], [21], [22].

In contrast to their essential role against respiratory fungal infection, neutrophils have been found to be dispensable for defense against the intracellular pathogens *Listeria monocytogenes* and *Toxoplasma gondii* [28], [29]. In both infection models, CCR2^+^Ly6C^hi^ inflammatory monocytes (CCR2^+^Mo, throughout this manuscript CCR2^+^Mo is used as an abbreviation for inflammatory monocytes, defined as CD45^+^CCR2^+^Ly6C^hi^CD11b^+^Ly6G^−^ leukocytes) were identified as essential innate effector cells that mediate bacterial and parasitic eradication [28], [29], [30], [31]. In these models, the formation of monocyte-derived TNF- and inducible nitric oxide synthase-producing dendritic cells (Tip-DCs) correlated with microbial clearance [31], [32], [33]. Since bacterial uptake by Tip-DCs during systemic listeriosis and salmonellosis appears to be an infrequent event (<1% of Tip-DCs) [34], [35], [36], it remains unknown whether inflammatory monocytes and their derivatives exert relevant antimicrobial activity by pathogen engulfment and killing at the portal of infection.

In fungal infection models, the role of CCR2^+^Mo has largely been understood in the context of adaptive CD4 T cell responses. In a respiratory *A.fumigatus* infection model CCR2^+^Mo are rapidly recruited to the lung and differentiate into CCR2^+^CD11c^+^MHCII^+^CD11b^+^CD103^−^ monocyte-derived DCs (Mo-DC) that are essential for the induction and maintenance of *A.fumigatus*-specific Th1 CD4 T cell responses [37], [38]. Mo-DCs have also been found to be important for initiation of fungus-specific T cell responses in the context *Blastomyces* vaccination and *Histoplasma capsulatum* infection in the lung [39], [40], [41], [42]. In vivo studies with human blood monocytes have shown that these cells have fungistatic activity ex vivo and elaborate cytokines and chemokines following stimulation with *A. fumigatus* conidia [43], [44], [45], [46]. Although emerging evidence indicates that CCR2^+^Mo and their derivatives contribute to innate defense against systemic candidiasis [47], [48], it remains unclear whether CCR2^+^Mo act to control the influx and activity of other effector cell populations or directly contribute fungicidal capacity at sites of infection.

One possible model is that CCR2^+^Mo and their derivatives control antifungal activity in the lung by regulating neutrophil influx, as suggested in LPS-induced models of pulmonary inflammation [49]. A second model of CCR2^+^Mo antifungal activity during respiratory fungal infection may involve the release of pro-inflammatory mediators [25] to enhance the fungicidal activity of resident or recruited leukocytes. A third model of antifungal activity involves direct antimicrobial effects of CCR2^+^Mo and derivative cells.

In the present study we set out to elucidate the mechanisms by which CCR2^+^Mo contribute to innate antifungal immunity in the lung. To this end, we employed genetically engineered mice that express a diphtheria toxin receptor (CCR2 depleter mice) or a GFP transgene (CCR2 reporter mice) under the control of the endogenous CCR2 promoter [29], [38] and fluorescent *Aspergillus* reporter (FLARE) conidia that trace the outcome of CCR2^+^Mo and Mo-DC interactions with conidia in the lung with single-encounter resolution [27]. We found that sustained depletion of CCR2^+^Mo and Mo-DCs led to the development of IA and a reduction in neutrophil conidiacidal activity. Beyond their impact on neutrophil conidiacidal responses, CCR2^+^Mo and Mo-DCs formed a TNF and iNOS-producing effector cell population in the lung that exerted rapid and effective conidiacidal activity similar in magnitude to neutrophil fungicidal activity. In aggregate, our studies suggest that CCR2^+^Mo and their derivatives mediate an essential role in antifungal defense in the lung by directly containing conidial germination and by enhancing neutrophil antifungal activity.

## Results

### CCR2^+^ inflammatory monocyte-depleted mice develop invasive aspergillosis

**Figure 1:**
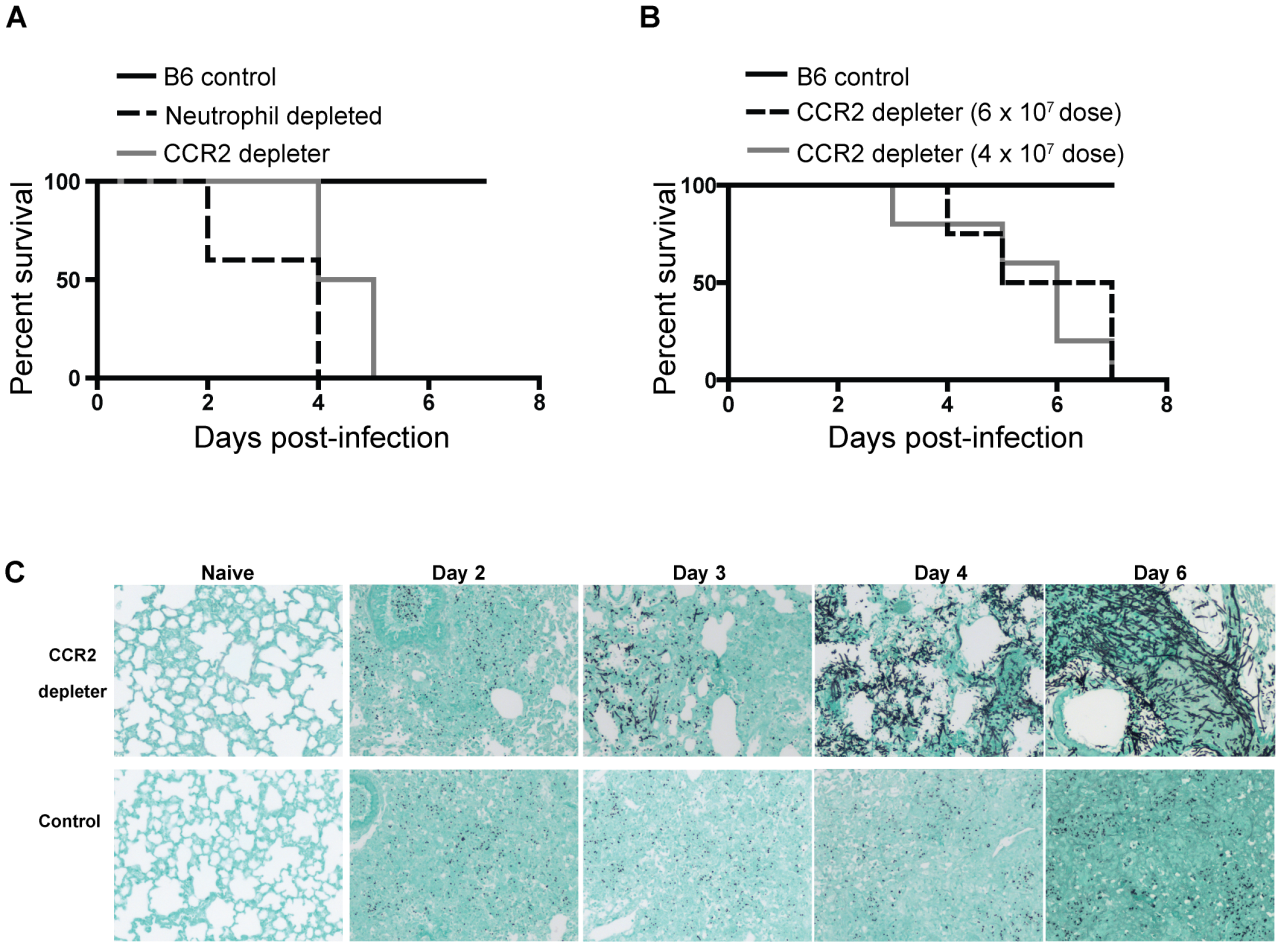
CCR2^+^ cells protect against Invasive Aspergillosis. A–B) CCR2 depleter (solid gray line) and control B6 non-transgenic littermates (solid black line) were treated with 250 ng of DT i.p. on day −1, +1, and +3. Neutrophil depleted mice (dashed black line) were B6 mice injected with 1A8 (anti-Ly6G antibodies) daily. (A) All animals were infected with 8×10^7^ live *A.fumigatus* conidia. The graph shows Kaplan-Meier survival of individual groups pooled from two independent experiments with 4–5 mice per group per experiment. Statistical analysis was performed with log-rank test and Bonferroni correction for multiple comparisons: WT vs. CCR2 depleter *P* = 0.0002, WT vs anti-Ly6G treated *P* = 0.0003. (B) Kaplan-Meier survival of DT-treated B6 (solid black line, inoculum 6×10^7^ conidia) and CCR2 depleter mice (6×10^7^ conidia, dashed black line; 4×10^7^ conidia, solid grey line). Statistical analysis was performed as described in (A). WT vs. CCR2 depleter 6×10^7^ p = <0.0001, WT vs CCR2 depleter 4×10^7^ p = 0.001. Data shown is for five mice per group. (C) Representative photomicrographs of formalin-fixed GMS-stained lung sections collected at the indicated times p.i. from DT-treated CCR2 depleter (top row) and B6 mice (bottom row). Naïve animals were sacrificed at day +6 and received 3 doses of DT. Sections shown are for one mouse per group and are representative of 3–5 mice that were examined per group per time point in two independent experiments.

To examine the contributions of CCR2^+^ Mo and their derivatives to respiratory fungal defense, we monitored the outcome of intratracheal *A. fumigatus* conidial challenge in CCR2 depleter mice [38] that express a functional diphtheria toxin receptor (DTR) under control of the CCR2 promoter. CCR2 depleter mice were treated with diphtheria toxin (DT) on day −1, +1, and +3 to ablate CCR2-expressing cells during respiratory fungal infection. We included two control groups: non-transgenic C57BL/6J (B6) littermates that received the same DT administration regimen as CCR2 depleter mice and B6 mice that were depleted of neutrophils by administration of anti-Ly6G antibodies. Consistent with previous studies using a different neutrophil-depleting antibody [20], [21], [22], anti-Ly6G-treated mice rapidly succumbed to IA (Figure 1A). Non-transgenic B6 control animals treated with DT did not develop disease symptoms throughout the duration of the experiment. Strikingly, CCR2 depleter mice treated with DT uniformly succumbed to infection when challenged with inocula that ranged from 4–8×10^7^ conidia (Figure 1A and 1B). To determine whether mortality was associated with fungal tissue invasion, Gomori methenamine silver (GMS)-stained lung sections were examined from CCR2 depleter mice and control animals at various time points post-infection. Lung sections from CCR2 depleter mice showed extensive and progressive hyphal growth (Figure 1C) starting at day +3 post infection (p.i). Extensive lung parenchymal destruction and obliteration of bronchoalveolar architecture was apparent at later time points. In contrast, lung sections from DT-treated B6 mice only showed evidence of conidia that failed to germinate at all time points examined. This is consistent with our previous studies in which B6 mice were able to effectively prevent conidial germination [37], [50], [51]. In aggregate these findings demonstrate that CCR2^+^ cells are essential for early host defense against *A.fumigatus* and that their ablation leads to the development of IA.

### Susceptibility of CCR2 depleter mice is not due to lack of NK cells

**Figure 2:**
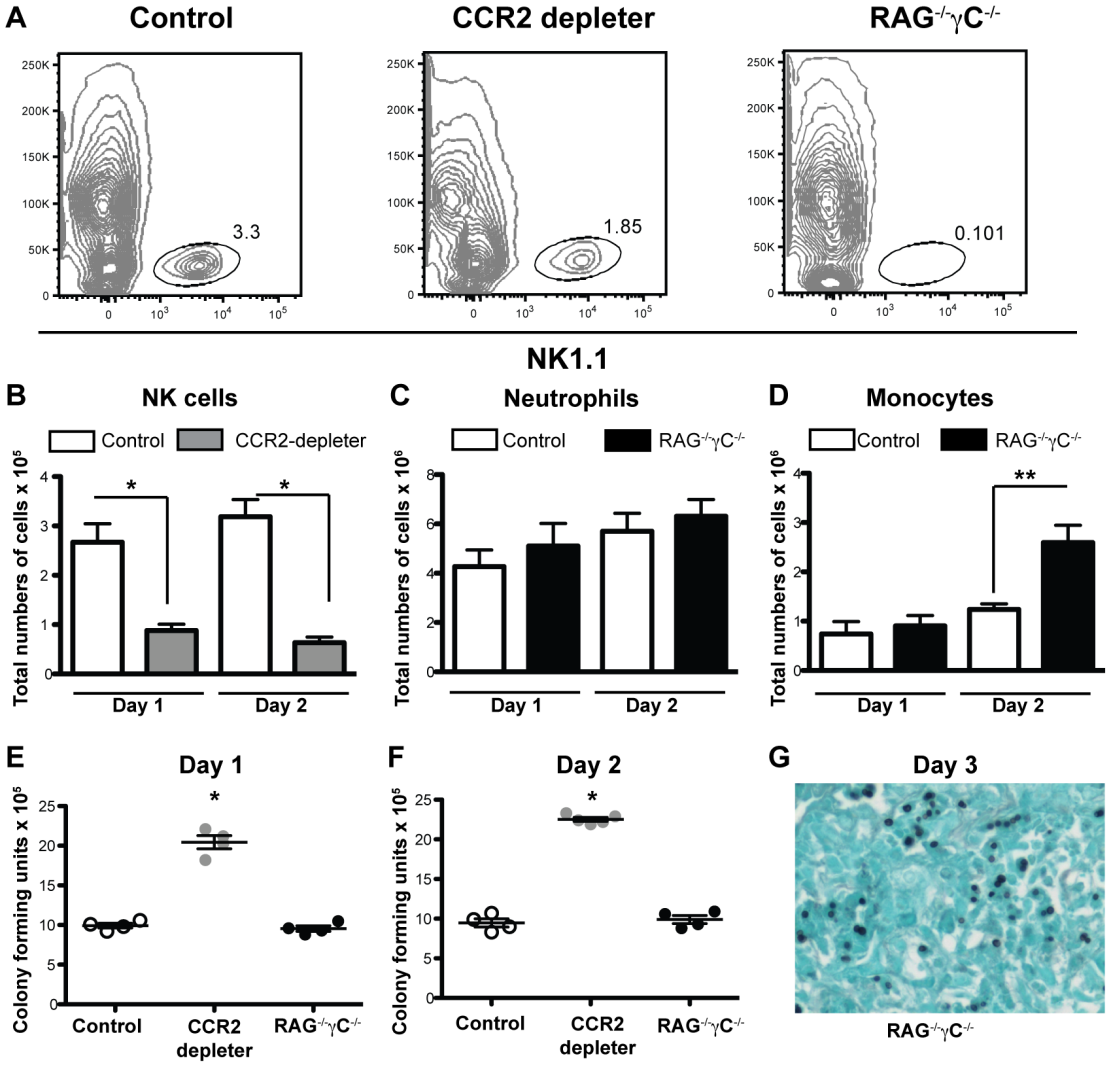
CCR2^+^ NK cells and innate lymphocytes are dispensable for innate defense against IA. (A) Representative plots of CD45^+^ lung cells obtained from control B6, DT-treated CCR2 depleter mice, and RAG^−/−^γC^−/−^ mice one day p.i. with 8×10^7^ *A.fumigatus* conidia and analyzed for NK1.1 expression. B–D) The bar graphs show the total number of lung (B) NK1.1^+^ cells, (C) CD11b^+^Ly6G^+^Ly6C^+^ neutrophils, or (D) CD11b^+^Ly6G^−^Ly6C^+^ monocytes (CCR2^+^Mo) in DT-treated CCR2 depleter (gray bars), control mice (white bars), or RAG^−/−^γC^−/−^ (black bars) at day +1 and +2 p.i. (E–F) The scatter plots show the mean ± SEM of lung CFUs recovered from control (white circles), DT-treated CCR2 depleter mice (gray circles) or RAG^−/−^γC^−/−^ (black circles) at day +1 and +2 p.i. (B–F) Data shown is for mean ± SEM for 4–5 mice per group from one of two representative experiments. Mann-Whitney test used for statistical analyses, * p<0.05, **p<0.01. G) The photomicrograph shows GMS-stained lung tissue from a representative RAG^−/−^γC^−/−^ mouse on day +3 p.i.

Previous studies have shown a protective role for NK cells in a neutropenic model of IA [24]. Since a subset of NK cells express CCR2, we explored whether the phenotype observed in CCR2 depleter mice could be linked to a defect in NK cells. We examined the recruitment of NK cells to the lung of CCR2 depleter mice and to control non-transgenic littermates during respiratory fungal infection. We observed that DT treatment significantly depleted NK cells in the lung of infected CCR2 depleter mice at 24 and 48 h p.i. (Figures 2A and 2B). To examine whether this reduction in NK cells could be linked to the development of IA in CCR2 depleter mice, we examined the progression of *A.fumigatus* infection in mice that lack all lymphocytes, including NK cells, iNKT cells, and innate lymphocytes (recombination activating gene [RAG-2] and common gamma chain [γC] double deficient mice; RAG^−/−^γC^−/−^). NK1.1^+^ cells were absent from the lungs of *A.fumigatus*-infected RAG^−/−^γC^−/−^ mice (Figure 2A) but the mice showed normal neutrophil and enhanced monocyte recruitment to infected lungs (Figures 2C and 2D). Despite a total lack of NK cells, RAG^−/−^γC^−/−^ mice controlled *A.fumigatus* conidial inocula at 24 and 48 h p.i., as judged by the recovery of viable fungal cells from the lungs of RAG^−/−^γC^−/−^ compared to control mice (Figures 2E and 2F). In addition, we did not observe invasive fungal growth in infected RAG^−/−^γC^−/−^ mice by lung histopathology (Figure 2G) and RAG^−/−^γC^−/−^ mice did not develop disease symptoms within the one week observation period. In contrast, CCR2 depleter mice showed a significant increase in the number of viable fungal cells in the lung at 24 and 48 h p.i. (Figures 2E and 2F) which preceded invasive fungal growth at 3 days p.i. (Figure 1C). In aggregate, these results indicate that the development of IA in CCR2-depleter mice cannot be explained by DT-induced ablation of CCR2^+^ NK cells.

### CCR2^+^Mo depletion and neutrophil recruitment during respiratory fungal infection

**Figure 3:**
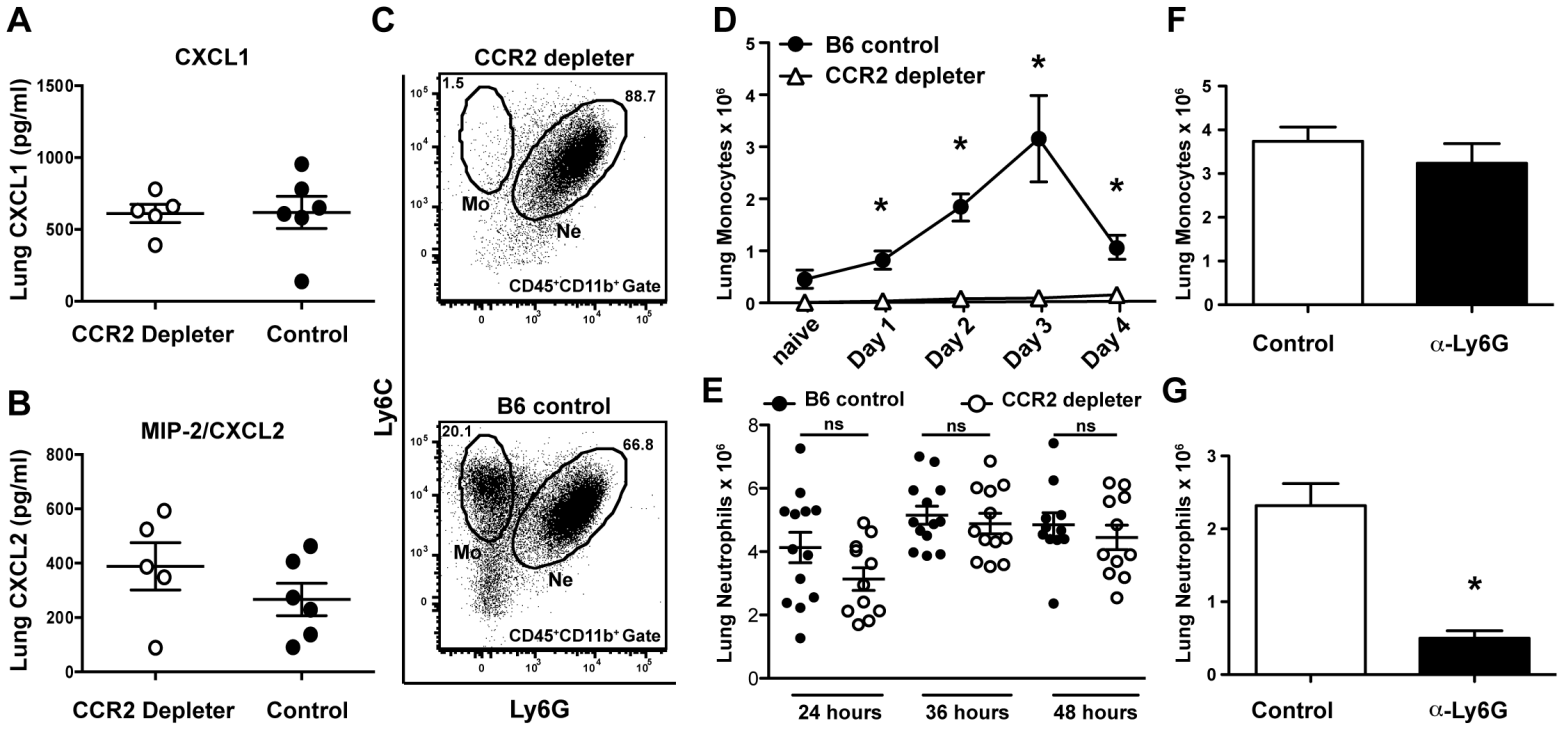
CCR2^+^ cells are dispensable for the production of neutrophil chemokines and neutrophil recruitment. (A–E) Control and CCR2 depleter mice were treated with DT and infected with 6×10^7^ conidia on day 0 and euthanized at the indicated times for ELISA of lung homogenates and FACS analysis of lung single cell suspensions. (A–B) The scatter plots show mean ± SEM lung (A) CXCL1 and (B) CXCL2 levels at 48 h p.i. in CCR2 depleter (white circles) and control B6 mice (black circles). (C–E) Representative FACS plots (day+1 p.i.) from CCR2 depleter (C, top row) and control B6 mice (C, bottom row) gated on lung CD45^+^CD11b^+^ cells and analyzed for Ly6C and Ly6G. Monocytes (Mo) are identified as Ly6C^+^Ly6G^−^ cells while neutrophils (Ne) are identified as Ly6G^+^Ly6C^+^cells. (D) The graph shows mean number (±SEM) of monocytes recovered from the lung of DT-treated B6 mice (black circles) or CCR2 depleter mice (white triangles) at the indicated time points p.i. Pooled data shown from three independent experiments (3–5 mice per group and per expt.). (E) The scatter plots show mean ± SEM of number of neutrophils recovered from the lung of CCR2 depleter mice (white circles) or control littermates (black circles) at various times after infection. Each symbol represents one mouse. Data is cumulative for two or three independent experiments with 3–5 mice per group per time point. (F–G) The bar graphs show the mean number (±SEM) of lung monocytes (F) and neutrophils (G) recovered from anti-Ly6G-treated and control mice as described in Figure 1. Statistical analyses were performed using Mann Whitney tests, n.s (not significant), * p<0.05.

Given the crucial role of neutrophils in defense against IA, we examined the impact of CCR2^+^Mo ablation on neutrophil chemotactic responses and recruitment to the lung. Although previous studies have clearly established that neutrophils are not directly eliminated by DT administration in CCR2 depleter mice [29], [38] we hypothesized that CCR2^+^Mo ablation could interfere with lung neutrophil recruitment due to their role as producers or amplifiers of chemotactic mediators, as has been observed in a LPS-induced model of lung inflammation [49]. To test this possibility, CCR2 depleter mice were treated with DT, infected with *A. fumigatus* conidia, and euthanized at various time points after infection to measure the production of neutrophil-recruiting chemokines and to enumerate and analyze lung homogenates by flow cytometry. CCR2 depleter mice treated with DT had similar lung levels of chemokine (C-X-C) motif ligand 1 (CXCL1) and CXCL2 as control non-transgenic littermates treated with DT, suggesting that CCR2^+^ cells are not required for the production of these chemokines during respiratory fungal infection (Figures 3A and 3B).

Although DT administration clearly eliminated all CCR2^+^Mo in infected mice (Figures 3C and 3D), DT administration did not deplete lung neutrophils (identified as CD45^+^CD11b^+^Ly6G^+^Ly6C^+^ cells) (Figure 3C). Furthermore, similar numbers of neutrophils were present in the lung of CCR2 depleter and control mice at various times after infection (Figure 3E). Although there was a modest trend towards lower numbers of neutrophils in CCR2 depleter mice these differences did not reach statistical significance. In contrast, B6 mice treated with anti-Ly6G antibodies had preserved lung CCR2^+^Mo recruitment (Figure 3F), but were depleted of neutrophils (Figure 3G). In aggregate, these findings indicate that CCR2 depleter mice produce wild-type levels of CXCL1 and CXCL2 during respiratory fungal infection and display preserved neutrophil recruitment to the site of infection, though these processes *per se* are insufficient to prevent the development of IA.

### Removal of CCR2^+^Mo impacts neutrophil conidiacidal activity

Since neutrophil recruitment was not affected by CCR2^+^Mo depletion, we hypothesized that neutrophil function may be altered, resulting in a reduction in neutrophil fungicidal activity in CCR2 depleter mice. To test this hypothesis, we utilized a recently developed fluorescent Aspergillus reporter strain (FLARE) to monitor and quantify neutrophil-mediated uptake and killing of *A.fumigatus* conidia in vivo [27]. The FLARE strain distinguishes live and dead conidia by incorporation of a tracer (Alexa Fluor 633; AF633) and a viability (DsRed) fluorophore. Host leukocytes that engulf live DsRed^+^AF633^+^ conidia emit two fluorescent signals, one of which (DsRed) is extinguished when leukocytes kill engulfed conidia. Using the FLARE strain, we quantified neutrophil conidial uptake and killing in CCR2 depleter and control mice.

**Figure 4:**
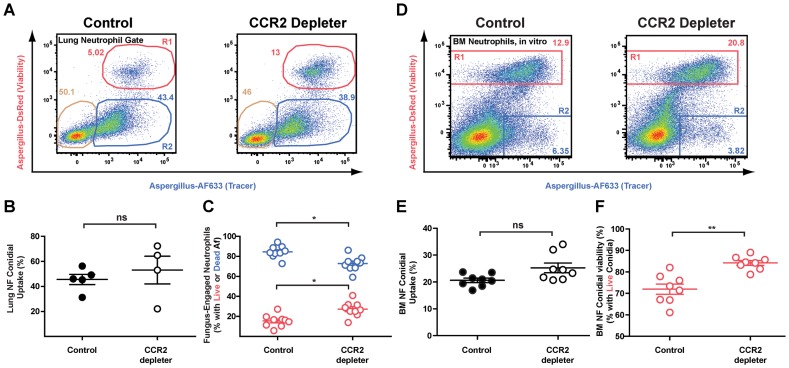
Diminished neutrophil conidiacidal activity in CCR2 depleter mice. CCR2 depleter and control mice were treated with 10/gm DT on day −1 and day 0 and infected with 3×10^7^ FLARE conidia. (A) Representative FACS plots of lung neutrophils isolated from CCR2 depleter mice and control mice and analyzed for dsRed and AF633 fluorescence. Plots show the frequencies of neutrophils that contain live (red gate) or killed conidia (blue gate) at 36 h p.i. (B) The scatter plots pooled from 2 experiments show the average frequency (± SEM) of lung neutrophil conidial uptake (R1+R2) and (C) lung neutrophil conidial viability (R1/(R1+R2) in CCR2 depleter and control mice. ^*^p<0.05 by Mann-Whitney test. (D) Representative FACS plots of bone marrow neutrophils isolated from control or CCR2 depleter mice and cultured in vitro with FLARE conidia. Neutrophils were identified as CD45^+^CD11b^+^Ly6G^+^ cells and analyzed for dsRed and AF633 fluorescence as shown. (E and F) The scatter plots pooled from 2 experiments show the average frequency (± SEM) of bone marrow in vitro neutrophil conidial uptake (R1+R2)(E) and in vitro conidial viability (R1/(R1+R2) in bone marrow neutrophils isolated from CCR2 depleter and control mice (F). ^**^p<0.01 by Mann-Whitney test.

Infection of DT-treated CCR2 depleter mice with FLARE conidia revealed that CCR2^+^Mo ablation did not alter the frequency of neutrophils with engulfed conidia at 12 or 36 hours p.i. compared to non-transgenic, DT-treated littermate controls (Figure 4B and data not shown), indicating that ablation of CCR2^+^Mo does not decrease neutrophil conidial uptake. However, the frequency of neutrophils with live conidia was substantially increased in DT-treated CCR2 depleter mice compared to control mice (Figures 4A and 4C). In other words, conidia engulfed by neutrophils were more likely to be killed in control mice than in CCR2 depleter mice (Figure 4C). Neutrophil expression of Toll-like receptor 2 and 4 and of the C-type lectin receptor Dectin-1 was similar in CCR2 depleter and in control mice at 36 p.i. (data not shown).

To extend these observations, we compared bone marrow neutrophil conidiacidal activity in vitro in the absence and presence of CCR2^+^ Mo, using bone marrow cells harvested from DT-treated CCR2 depleter and non-transgenic littermate controls. When CCR2^+^ Mo were absent from neutrophil–conidia co-culture experiments, neutrophil conidial viability was higher than in co-cultures that included CCR2^+^ Mo, though neutrophil conidial uptake was similar in both cases (Figures 4D–4F). Addition of flow-sorted bone marrow monocytes (identified as CCR2^(GFP+)^, CD11b^+^CD11c^−^NK1.1^−^ cells) restored the conidiacidal function of neutrophils to baseline levels (Figure S1). These findings indicate that CCR2^+^ Mo and derivative cells enhance neutrophil conidiacidal activity when these leukocytes are combined as purified cellular components in the test tube or are found in the complex inflammatory context within the lungs.

### CCR2^+^Mo differentiate into Mo-DCs that produce a variety of protective factors during respiratory fungal infection

**Figure 5:**
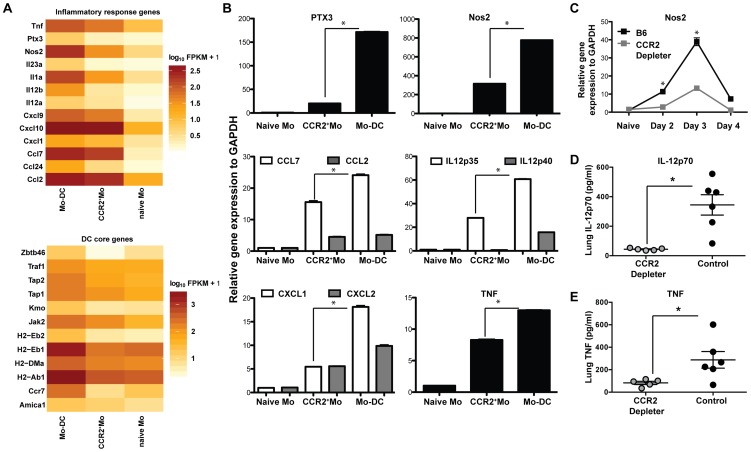
Inflammatory responses of CCR2^+^Mo and Mo-DC during respiratory fungal infection. Lung CCR2^+^Mo (GFP^+^CD45^+^CD11b^+^CD11c^−^Nk1.1^−^) and Mo-DC (GFP^+^CD45^+^CD11b^+^CD11c^+^NK1.1^−^) were FACS sorted 48 h p.i. from CCR2 reporter mice (purity >97% for all sorts) for transcriptome analysis by RNA-seq (A) or for quantitative RT-PCR (B). Control CCR2^+^Mo were also isolated from the lung of uninfected CCR2 reporter mice (naïve sample) to >97% purity. (A) Gene expression data shown in A is for one experiment and representative of 3 independent biological replicates and three idependent sequencing reactions using SOLiD sequencing platform. Differences in gene expression are shown as fragments per kilobase (FPKM) as calculated using Cufflinks and R software. (B) The graphs show expression of specific transcripts in the indicated cell populations by qRT-PCR using Taq-Man probes normalized to GAPDH. Data shown is mean ±SEM pooled from two separte experiments. (C) The graph shows pulmonary Nos2 induction in DT-treated CCR2 depleter and control mice at the indicated time points p.i. Data shown is mean ±SEM pooled from two separte experiments with 3 mice per group per time point. (D–E) The scatterplots show mean ± SEM lung (D) IL-12p70 and (E) TNF levels at 48 h p.i. in CCR2 depleter (grey circles) and control B6 mice (black circles) as in Figure 3A.

To determine additional mechanisms by which CCR2^+^Mo and/or Mo-DC mediate protection against *A.fumigatus*, we performed a transcriptome analysis on sorted cell populations with RNA-seq. To this end we infected CCR2 reporter mice with *A.fumigatus* and sorted CCR2^+^Mo and Mo-DC (identified as CCR2^(GFP+)^, CD11b^+^CD11c^+^NK1.1^−^) 48 h p.i. to >97% purity. CCR2^+^Mo present in the lung of naïve CCR2 reporter mice were sorted as a control population. We performed three independent experiments and found consistent upregulation of multiple cytokines and chemokines in response to fungal infection (Figure 5A), with the highest expression of these genes in the Mo-DC subset, as confirmed by qRT-PCR (Figure 5B). Cells isolated in the GFP^+^CD11b^+^CD11c^+^ fraction expressed genes identified as part of the core DC signature (Figure 5A) [52], consistent with their designation as dendritic cells (Mo-DCs). CCR2^+^Mo and Mo-DCs were not only capable of producing IL-12, Nos2 and TNF upon infection but appeared to act as essential sources for these inflammatory mediators during respiratory fungal infection, since ablation of these cells in CCR2 depleter mice resulted in significantly diminished production of these factors (Figures 5C–E). These findings thus suggest that CCR2^+^Mo and Mo-DC recruited to the lung during *A.fumigatus* infection express soluble factors, including cytokines (e.g. TNF) and effector molecules (e.g. pentraxin-3) that enhance neutrophil antifungal activity.

### CCR2^+^Mo and Mo-DCs are required for direct fungal spore elimination

To examine whether CCR2^+^Mo and Mo-DCs play a direct role in conidial killing we infected CCR2 reporter with FLARE conidia to track the dynamics of pulmonary CCR2^+^Mo recruitment, their differentiation into Mo-DC, and their conidiacidal activity. CCR2^+^ cells in the lung are comprised primarily of CCR2^+^CD11b^+^Ly6C^+^ inflammatory monocytes (CCR2^+^Mo) that are present in the naïve lung (Figure S2) and are rapidly recruited from bone marrow stores during respiratory fungal infection [38]. CCR2^+^Mo rapidly upregulate CD11c and MHC class II expression levels in the inflamed lung (Figure S2, [38]).

**Figure 6:**
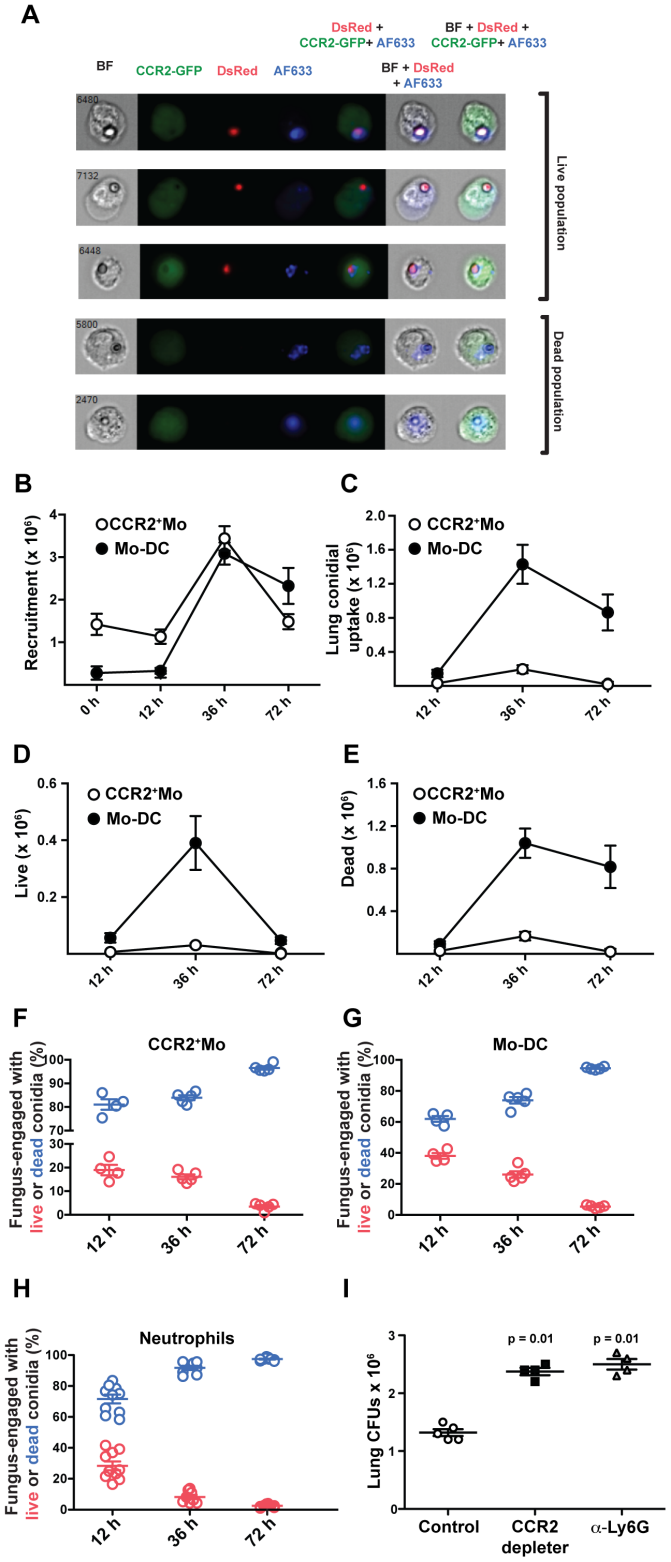
CCR2^+^Mo differentiate into Mo-DC and efficiently kill *A.fumigatus*. CCR2 reporter mice were infected with FLARE conidia and lung cell suspensions were enumerated and examined by (A) imaging cytometry and (B–H) flow cytometry. (A) Imaging cytometry of lung GFP^+^ (CCR2^+^) cells from FLARE-infected CCR2 reporter mice 36 h p.i. The micrograph depicts dsRed^+^AF633^+^ and dsRed^−^AF633^+^ monocytes that contain live and killed conidia, respectively. BF, bright-field. (B–E) The graphs show the total number (mean ± SEM) of lung CCR2^+^Mo (white circles) and Mo-DCs (black circles) at the indicated times p.i. . CCR2^+^Mo (white circles), and Mo-DC (black circles) were identified as shown in Figure S1. (B) Data shows total recrutiment of each subset over time. (C) The graph shows the total number of CCR2^+^Mo (white circles) or Mo-DCs (black circles) that contain engulfed conidia. (D–E) The graph shows the total number of CCR2^+^Mo (white circles) or Mo-DCs (black circles) that contain (D) live or (E) killed conidia. (F–H) Comparison of CCR2^+^Mo, Mo-DC and neutrophil conidiacidal activity. The scatter plots show the frequency of fungus-engaged (F) CCR2^+^Mo, (G) Mo-DC, and (H) neutrophils that contain live (red circles) or killed (blue circles) FLARE conidia at the indicated times p.i. Results are pooled from two experiments. (I) The graph shows lung CFUs from DT-treated B6 controls (white circles), DT-treated CCR2 depleter (black squares), and anti-Ly6G-treated B6 mice (grey triangles) at day +1 p.i.. Each symbol represents one mouse. Results are for one experiment representative of two individual experimenst for all data shown in this figure. Statistical analysis was performed using a Mann Whitney test.

To determine whether CCR2^+^Mo and Mo-DCs are capable of conidial killing in vivo, we first performed imaging cytometry of GFP^+^ cells isolated from FLARE-infected CCR2 reporter mice. We found evidence of GFP^+^ cells that contain viable DsRed^+^AF633^+^ conidia as well as GFP^+^ cells that contain killed AF633^+^ conidia (Figure 6A). To define the relative contribution of CCR2^+^Mo and their derivative Mo-DCs to conidial killing in vivo, we determined the kinetics of cell recruitment (Figure 6B), conidial uptake (Figure 6C), and killing by flow cytometry (Figures 6D and 6E). This analysis revealed that although similar numbers of CCR2^+^Mo and Mo-DCs were present in the lung at 36 h p.i. (Figure 6B), Mo-DCs were far more likely to engulf conidia and contain killed conidia compared to CCR2^+^Mo (Figures 6C and 6E).

Analysis of conidiacidal activity on a per cell basis revealed that once conidia were internalized, CCR2^+^Mo and Mo-DCs were as efficient in mediating conidial killing as neutrophils (Figures 6F–6H). The efficiency of conidial killing was determined by examining different fungus-engaged leukocyte populations (neutrophils, CCR2^+^Mo, Mo-DCs) and by comparing the frequencies of fungus-engaged leukocytes that contain either viable conidia or killed conidia. To examine the requirement for NADPH oxidase in Mo-DC conidiacidal activity, we generated mixed bone marrow chimeric mice that contained equal numbers of congenically marked NADPH oxidase-deficient (p47phox^(−/−)^ and –sufficient (p47phox^(+/+)^) hematopoietic cells. In this host setting, NADPH oxidase-deficient and –sufficient leukocytes are isolated from and analyzed in the same inflammatory context. Similar to neutrophils, Mo-DCs employ reactive oxygen species (ROS) as a conidiacidal mechanism, since NADPH-deficient Mo-DCs kill conidia less effectively than NADPH-oxidase sufficient counterparts (Figure S3). Analysis of FLARE killing by Mo-DCs showed that the frequency of viable conidia in p47^phox−/−^ Mo-DCs was higher compared to p47^phox+/+^ Mo-DCs (Figure S3). Despite the superior conidiacidal acitivity in p47^phox+/+^ Mo-DCs, there was significant killing preserved in p47^phox−/−^ cells, indicating that conidial killing by Mo-DCs is only partially dependent on NADPH oxidase. When neutrophils and Mo-DCs were analyzed side-by-side, neutrophil conidiacidal activity was more dependent on NADPH oxidase activity than Mo-DC conidiacidal activity (data not shown and [27]). These findings indicate that Mo-DCs, similar to neutrophils, employ NADPH oxidase activity as a conidiacidal mechanism.

The total number of viable fungal cells in the lung of CCR2 depleter mice was significantly elevated at (Figure 6I), demonstrating that lung conidiacidal activity is significantly reduced at early time points p.i. when CCR2^+^Mo and Mo-DCs are ablated, consistent with an essential role in innate antifungal defense in the lung. Although essential, CCR2^+^Mo and Mo-DCs *per se* are not sufficient for conidial containment since monocyte-sufficient, neutropenic mice (anti-Ly6G treated mice) also showed enhanced conidial survival and fungal germination in the lung (Fig. 6I). In aggregate our findings are consistent with a model in which CCR2^+^Mo and Mo-DC derivatives are essential in preventing IA development via a non-redundant role in conidial clearance by direct killing and by regulation of neutrophil conidiacidal activity.

## Discussion

In this study, we uncover novel and essential functions for CCR2^+^ inflammatory monocytes and their derivative Mo-DCs in innate antifungal defense in the lung. The protective role of CCR2^+^Mo and their derivatives against *A. fumigatus* is not compensated by neutrophil antifungal activity. Similarly, our findings confirm the long-standing tenet that neutrophil function is essential for host defense against IA [19], [20], [21], [22]. Thus, CCR2^+^Mo and derivative Mo-DC as well as neutrophils represent essential innate immune cells that prevent the formation of tissue-invasive hyphae and IA in the murine lung. In contrast, NK cells and other common gamma chain-dependent innate lymphocyte populations were not essential to mediate innate defense against inhaled *A. fumigatus* conidia, since mice deficient in these leukocyte populations contained conidial germination and did not develop invasive disease.

Previous studies showed that neutrophil depletion leads to increased pulmonary recruitment of CD11b^+^CD11c^+^ TNF-producing DCs [25]. The TNF-producing DC population described by Park et al. [25] was recruited in response to enhanced CCL2 production and appears similar to Mo-DCs described in our study. The finding that ablation of CD11c-expressing cells diminished fungal clearance in this model was consistent with a protective role of one or several CD11c-expressing myeloid cell subsets in the context of neutropenia [25]. Similarly, the accumulation of CD11b^+^CD11c^+^ myeloid DCs in the lung was greater in CCR7^(−/−)^ neutropenic mice than in CCR7^(+/+)^ neutropenic mice. This finding correlated with reduced susceptibility to IA, consistent with a protective role of CD11b^+^CD11c^+^ DCs at the site of respiratory fungal infection in neutropenic animals [53].

In our experiments, we examined the relationship between CCR2^+^ Mo and their derivatives and neutrophil recruitment and function in the lung. DT-treated CCR2 depleter and control mice showed similar kinetics and magnitude of neutrophil recruitment during the early phases of infection. In the respiratory *A. fumigatus* infection model, conidial clearance is a hallmark of the first 24 hours post-infection. In both mouse strains, the number of viable fungal cells is reduced by a factor of five to ten during this time period, with a more effective reduction in monocyte-sufficient mice compared to monocyte-depleted mice. This early difference in conidial clearance occurs despite the preserved synthesis of neutrophil chemotactic factors in the lung and the rapid accumulation of neutrophils at the site of infection. Thus, the difference in fungal CFUs among the groups likely reflects three factors: the early absence of CCR2-Mo and derivative cell conidiacidal activity, the reduction in neutrophil conidiacidal activity on a per-cell basis, and neutrophil recruitment that may be considered suboptimal since the number of viable fungal cells in the lung of CCR2 depleter mice is on average twice as high as in control mice.

Essential protective functions for monocyte-derived DCs subsets have been demonstrated in other infection models [28], [30], [31], [54], [55]. In the context of systemic *Listeria monocytogenes* infection CCR2-dependent, TNF- and iNOS-producing DCs (Tip-DC) were found to play an essential role in innate defense against intracellular bacteria [31], a finding that has been extended to other intracellular pathogens, for example *Leishmania* and *Toxoplasma* [28], [30], [55], [56]. Our studies now show that the protective function for CCR2^+^Mo and their derivative cells is not restricted to intracellular bacteria and parasites but is also essential for innate antifungal defense. In response to *A. fumigatus* infection, CCR2^+^ Mo-DCs produced TNF and iNOS and are likely comparable to Tip-DCs induced by *L. monocytogenes* infection. Given the unique composition of fungal pathogens it will be important to examine how the recruitment and differentiation of CCR2^+^Mo into Tip-DCs is regulated by innate receptors specialized in fungal recognition.

During systemic listeriosis, Tip-DCs mediate protective effects due to their role as major producers of TNF and nitric oxide [31]. The association of defects in TNF signaling with murine susceptibility to IA and of TNF inhibitors with human susceptibility to IA indicates that TNF plays a critical role in antifungal immunity in the lung. Although CCR2^+^Mo are a major source of TNF early during respiratory *A. fumigatus* infection, the precise function of TNF during conidial clearance remains to be established. It is unclear whether TNF-producing CCR2^+^Mo represent an important target of TNF signaling to enhance cell-intrinsic conidiacidal activity. In an ocular model of fungal keratitis, iNOS activity was dispensable for host defense against *A. fumigatus* in the cornea. The role of CCR2^+^Mo-derived nitric oxide during pulmonary fungal infection remains undefined. In both instances, the development of cell type-specific gene knockout strategies [57] will enable researchers to address these questions.

Besides their role as producers of inflammatory mediators our data shows that CCR2^+^ Mo and Mo-DCs are crucial for direct conidial containment. Although both populations kill conidia efficiently, the frequency of Mo-DCs with engulfed conidia is far higher than that of CCR2^+^Mo. Thus, Mo-DCs kill a significant larger number of conidia than CCR2^+^ Mo. Unlike alveolar macrophages [58], the conidiacidal activity of CCR2^+^Mo and their derivatives was partially dependent on NADPH oxidase. Thus, CCR2^+^Mo and their derivatives contribute to ROS-dependent mechanisms that are implicated in human defense against *Aspergillus sp.*, i.e. the susceptibility of patients with chronic granulomatous disease to IA. [23], [26], [59]. These findings are similar to observations in leishmaniasis, in which CCR2^+^Mo mediate elimination of parasites via the production of reactive oxygen species (ROS) [55]. During secondary responses to *L. monocytogenes* infection, inflammatory monocytes also represent a significant source of protective ROS [60].

Our work extends previous studies on the role of CCR2^+^Mo and their derivatives in trafficking fungal antigen to lung-draining lymph nodes, priming *Aspergillus*-specific CD4 T cells, and in inducing the development of Th1 effector cells. Taken together, our findings suggest that CCR2^+^Mo are required in antifungal defense as innate conidiacidal effectors and precursors of inflammatory Mo-DCs; the latter cells provide a significant reservoir of conidiacidal activity in the lung and elicit Th1 responses [37], [38] that perpetuate a protective immune response [37]. Whether lung-resident *Aspergillus*-elicited Tip-DCs described in this study are identical to migratory Mo-DCs required for fungus-specific CD4 T cell priming is not clear at this time. It is possible that a subset of Tip-DCs migrate to the lung-draining lymph node for antigen transport and CD4 T cell priming or that a subset of Mo-DCs that do not produce TNF and iNOS are responsible for fungal antigen trafficking. Further studies will be required to dissect these possibilities.

Although the current study addresses the role of inflammatory monocytes in a murine model, it is possible that human monocytes similarly carry out an important role in defense against IA. The antifungal capacity of human monocytes against *A.fumigatus* has long been recognized [46] and exogenous cytokines, including M-CSF, IFN-γ and IL-12, enhance antifungal effects of these cells in vitro [46], [61], [62]. More detailed analysis of human monocyte subsets showed that CD14^+^CD16^−^ monocytes could prevent conidial germination [45]. In contrast, CD14^+^CD16^+^ monocytes mounted robust inflammatory responses to conidia but did not prevent germination in vitro [45], suggesting distinct contributions of human monocyte subsets to antifungal defense. Interestingly, CD14^+^CD16^−^ monocytes express CCR2 and have been proposed to be analogous to murine CCR2^+^ Mo [63]. Thus, the direct conidiacidal activity observed in murine CCR2^+^ Mo and their derivatives in the lung is likely functionally conserved in human CD14^+^CD16^−^ monocytes and their derivatives. In human neutropenic pulmonary aspergillosis there is significant pulmonary recruitment of CD1a^+^ DCs, which represent monocyte-derived cells [25].

Patients with autosomal dominant or sporadic deficiency in monocytes, DCs, and NK cells (termed MonoMAC syndrome) due to mutations in the transcription factor GATA2 are prone to disseminated nontuberculous mycobacterial infections (incidence ∼80%), invasive fungal infections (incidence ∼30%), primarily histoplasmosis but also aspergillosis, and to viral infections (e.g. human papilloma virus; incidence ∼80%). The clinical manifestations of patients with MonoMAC syndrome support the notion that circulating myeloid cells, independent of neutrophils and tissue-resident macrophages, play an essential role in antifungal defense [64], [65], [66]. The ablation of circulating monocytes and monocyte-derived DCs as well as the partial loss of NK cells in CCR2 depleter mice is similar to the quantitative defects in circulating monocytes, DCs, and NK cells observed in MonoMAC patients and in both instances, hosts are vulnerable to invasive fungal disease (this work and [21]). Although neutropenia has long been considered the most important risk factor for IA development in patients with hematologic malignancies and in allogeneic HCT patients, there is clinical evidence that monocytopenia represents an additional risk factor for IA development [67], [68]. In aggregate, these lines of evidence suggest that the importance of CCR2^+^Mo in antifungal defense is likely not exclusive to murine models of IA, but reflective of a conserved essential function of these cells in antifungal defense.

## Materials and Methods

### Mice

The CCR2 depleter (CCR2-DTR) and CCR2 reporter (CCR2-GFP) strains were generated on the C57BL/6 background as previously described [38], [69]. Control animals for CCR2^+^Mo-depletion experiments were sex and age-matched, non-transgenic littermates. For antibody depletion experiments, sex and age-matched C57BL/6 mice were purchased from Jackson Laboratories. RAG^−/−^γC^−/−^ (RAG-2^−/−^IL2rg^−/−^) lymphopenic mice were purchased from Taconic. All strains were maintained and bred in the Rutgers-NJMS Cancer Center Research Animal Facility or in the Fred Hutchinson Cancer Research Center Animal Health Resources Facility under specific pathogen-free conditions. Mixed bone marrow chimeric mice were generated as described in (Jhingran et al., 2012) [27] by transferring an equal mixture of CD45.1^+^ p47phox^(+/+)^ and CD45.2^+^ p47phox^(−/−)^ bone marrow cells using lethally irradiated CD45.1^+^CD45.2^+^ recipients. Recipient mice were rested for 6 weeks prior to experimental infection. Animal studies were performed following biosafety level 2 (BSL-2) protocols approved by the Institutional Animal Care and Use Committee (IACUC) of Rutgers University and of Fred Hutchinson Cancer Research Center.

### Infection, culture, and histology

For these studies, we employed an *Aspergillus fumigatus*-DsRed expressing strain (Af293.1RFP) [70], a generous gift from Dr. Michelle Momany. For lung ELISA studies, we used *Aspergillus fumigatus* strain Af293. *A. fumigatus* was cultured on Sabouraud dextrose agar (SDA) for 7–10 days prior to infection. Mice were challenged with 4–8×10^7^ live conidia per mouse using a non-invasive intratracheal (i.t.) infection procedure as previously described [51]. The viability of *A. fumigatus* conidia in the inoculum was confirmed by plating serial dilutions on SDA. For assessment of fungal burden in infected mice lung single-cell suspensions were serially diluted and plated on SDA at various times after infection. For histological examination, lungs were perfused with 10 ml of PBS to remove blood and fixed in 10% buffered formalin. Fixed lung tissue was paraffin embedded and stained with modified GMS stain at the Histology Core Facility (Rutgers-NJMS).

### Cell depletion strategies

For the selective removal of neutrophils, mice were injected daily with 1A8 monoclonal antibodies (anti-Ly6G). Mice were injected with 500 µg i.p together with another dose of 100 µg i.t of 1A8 antibodies in order to achieve significant depletion of Ly6G^+^ neutrophils in the lung as previously reported [71]. Highly concentrated, purified 1A8 antibodies were isolated from ascites fluid following IACUC approved protocols (Rutgers-RWJMS). For depletion of CCR2^+^ cells, CCR2-DTR mice and control CCR2-DTR negative littermates received 250 ng of diphtheria toxin i.p. one day prior to infection and every other day thereafter in order to maintain depletion. Diphtheria Toxin was purchased from List Biological Laboratories (Campbell, CA), and reconstituted at 1 mg/ml in PBS. Aliquots were stored in −80°C. The specificity and efficiency of depletion in the lung was confirmed by flowcytometric analysis.

### Lung cell isolation and flow cytometry

Lung samples were minced in PBS with 3 mg/ml collagenase type IV (Worthington), and were incubated at 37°C for 45 min to obtain single cells suspensions. After digestion, lung suspensions underwent RBC lysis. All antibodies were purchased from BD Biosciences. The staining protocols included combinations of the following antibodies: Gr-1 (RB6-8C5 FITC), Ly6C (AL-21 PE), Ly6G (1A8 APC), CD11b (M1/70, PerCP Cy5.5), CD11c (N418 Pacific Blue), MHC Class II I-A/I-E (M5/11.415.2, Alexa Fluor 700), and CD45 (30-F11 APC-Cy7). Samples were collected zon a BD LSRII Flow Cytometer and analyzed using FlowJo software.

### Analysis of cytokines and RNA expression in lung tissue

Total RNA from lungs was extracted with Trizol (Invitrogen). Relative mRNA levels were determined by qRT-PCR. One microgram of total RNA was reverse transcribed using High Capacity cDNA Reverse Transcription Kit (Applied Biosystems). Taq Man Fast Universal PCR Master Mix (2×) No Amp and TaqMan probes (Applied Biosystems) for each gene were used, and normalized to GAPDH. Gene expression was calculated using ΔΔCT method relative to naïve sample. For cytokine and chemokine measurements we performed ELISAs on lung homogenates according to the manufacturer's instructions. Mouse CXCL1 and CXCL2 ELISA kits were purchased from R&D systems. IL-12p70 and TNF ELISAs were obtained from BD Bioscience.

### Cell Sorting, RNA sequencing and analysis

CCR2GFP^+^CD45^+^CD11b^+^NK1.1^−^CD11c^−^ (CCR2^+^Mo) and CCR2^+^CD45^+^CD11b^+^NK1.1^−^CD11c^+^ (Mo-DC) populations were isolated to more than 97% purity using a BD FACS ARIA II cell sorter dedicated for the processing of BSL-2 samples (Flowcytometry core facility NJMS). Cell subsets were sorted from lung single cell suspensions obtained from *A.fumigatus*-infected CCR2-GFP mice that were challenged 2 days earlier. CCR2GFP^+^CD45^+^CD11b^+^NK1.1^−^CD11c^−^ cells (Mo-naïve) were sorted from uninfected CCR2-GFP mice. DAPI was used as a viability control during sort. Immediately after sorting RNA was extracted using Qiagen RNeasy kit. One microgram of total RNA was rRNA depleted using the Ribominus Human/Mouse depletion module. Library generation and sequencing was performed by the Molecular Resource Facility at Rutgers-NJMS. Briefly, The SOLiD™ Total RNA-Seq Kit (P/N 4445374) was used to convert rRNA-depleted RNA into a cDNA library for analysis on the Applied Biosystems SOLiD™ Sequencing System. The RNA was fragmented using RNase III to produce 100 to 300 base fragments which were then size selected and purified using the Purelink RNA micro kit (Applied Biosystems, Foster City, CA). The yield and size distribution of fragmented RNA was confirmed using the RNA 6000 Pico Chip kit on a Bioanalyzer (Agilent Technologies, Santa Clara, CA). The fragmented RNA was hybridized and ligated to Solid™ oligonucleotide adaptors and RNA ligation reagents. Reverse transcription was done using ArrayScript Reverse Transcriptase to generate the cDNA which is was then purified and size-selected using Agencourt® AMPure® XP Reagent (Beckman Coulter, Inc., Brea, CA), to ensure capture and size-selection of cDNA greater than 150 bp. The cDNA was amplified and purified using Invitrogen Purelink PCR Micro kit. The library size and concentration was confirmed using the Bioanalyzer DNA1000 kit and was used to generate template for sequencing using emulsion PCR. Three independent cell sorting and RNA sequencing reactions were performed. RNA seq results of representative genes were confirmed by qRT-PCR. The SOLiD reads were aligned to the mm9 mouse reference genome using Tophat [72] 2.0.8b and expression levels were determined using Cufflinks [73] 2.1.1 and the UCSC genome annotation.

### Analysis of in vivo and in vitro conidial uptake and killing

FLARE conidia were generated as described in [27]. Briefly, to generate FLARE conidia, 5×10^8^ Af293-dsRed conidia were rotated in 0.5 mg/ml Biotin XX, SSE (B-6352; Invitrogen) in 1 ml of 50 mM carbonate buffer (pH 8.3) for 2 hr at 4°C and labeled with 0.02 mg/ml AF633-streptavidin (S-21375; Invitrogen) in 1 ml PBS for 30 min at RT, and resuspended in PBS and 0.025% Tween 20 for use within 24 h. In all experiments, leukocyte conidial uptake refers to the frequency of fungus-engaged neutrophils (dsRed^+^AF633^+^+dsRed^−^AF633^+^). Conidial viability within a specific leukocyte subset refers to the frequency of leukocytes that contains live conidia (dsRed^+^AF633^+^) among all fungus-engaged leukocytes of the particular subset. For in vitro studies of neutrophil conidiacidal activity, neutrophils were isolated from the bone marrow of CCR2 depleter mice treated with DT for 24 hours or from DT-treated transgene-negative, littermate controls. Bone marrow cells were obtained by flushing the femurs and tibia bone cavities with PBS. Bone marrow cell suspensions were enriched for neutrophils using a density gradient-centrifugation protocol as described by Swamydas, et al [74]. BM neutrophils were cultured in the presence or absence of monocytes together with FLARE conidia at a multiplicity of infection of 1∶4 conidia to cell ratio. FLARE conidia killing was assessed at 24 hours post culture initiation as described above. For in vitro reconstitution, BM neutrophils were FACS sorted from BM of CCR2 depleter mice and cultured in the absence or presence of BM monocytes that were FACS sorted from CCR2-GFP as GFP^+^CD11b^+^Ly6C^+^Ly6G^−^NK1.1^−^ cells. Monocytes were cultured at 1∶4 ratio relative to neutrophils numbers to reflect the ratios of these cells seen in vivo.

### Ethics statement

The studies performed were governed by protocol 10094E1213 as approved by the IACUC committee of New Jersey Medical School and by protocol 1813 as approved by the IACUC committee at the Fred Hutchinson Cancer Research Center. Animal studies were compliant with all applicable provisions established by the Animal Welfare Act and the Public Health Services (PHS) Policy on the Humane Care and Use of Laboratory Animals.

## Supporting Information

Figure S1 **Killing of neutrophils isolated from CCR2 depleter mice is restored by culture with monocytes in vitro.** Neutrophils were FACS sorted from the bone marrow of CCR2 depleter mice treated with DT and cultured alone or together with sorted monocytes. Monocytes were FACS sorted from the bone marrow of CCR2-GFP reporter mice and cultured with neutrophils at 1∶4 Mo∶NF ratio. FLARE conidia were added at 1∶4 conidia∶cell ratio. Scattered plots from an experiment show the average frequency (± SEM) of conidia viability within the neutrophil gate examined 24 hours after culture initiation. Statistical analysis was done by Mann-Whitney test.
(TIF)

Figure S2 **CCR2^+^Mo rapidly differentiate into Mo-DCs in response to** ***A.fumigatus*** **infection.** CCR2-GFP reporter mice were infected with live *A.fumigatus* conidia and cell recruitment to the lung was examined at the indicated times. FACS plots are for one representative mouse. Top row: plots were gated on CD45^+^ cells, middle row: plots are gated on gates shown on top row, bottom row: MHC class II expression in populations A, B and C as gated on middle row panels. Data is representative of two independent experiments.
(TIF)

Figure S3 **NADPH Oxidase mediates Mo-DC-dependent conidial killing in the lung.** BM chimeric (1∶1 mix of CD45.1^+^ p47^phox(+/+)^ and CD45.2^+^ p47^phox(−/−)^ BM cells into irradiated CD45.1^+^CD45.2^+^ recipients) were infected with 3×10^7^ FLARE conidia. (A) Representative plots of p47^phox(+/+)^ and p47^phox(−/−)^ CD11b DCs (CD45^+^MHCII^+^CD11c^+^CD103^−^CD11b^+^) analyzed for dsRed and AF633 fluorescence show the frequencies of CD11b DCs that contain live (red gate) or killed (blue gate) conidia 36 h p.i. (B and C) Scattered plots from an experiment show the average frequency (± SEM) of CD11b DC (B) conidial uptake (R1+R2) and (C) conidial viability (R1/(R1+R2) in p47^phox(+/+)^ and p47^phox(−/−)^ cells. ^*^p<0.05 by paired t-test.
(TIF)
