# Supplementary figures and images for: Inflammatory Monocytes Orchestrate Innate Antifungal Immunity in the Lung
Source: PLoS Pathog. 2014 Feb 20;10(2):e1003940. doi: 10.1371/journal.ppat.1003940 (PMC3930594; doi:10.1371/journal.ppat.1003940)

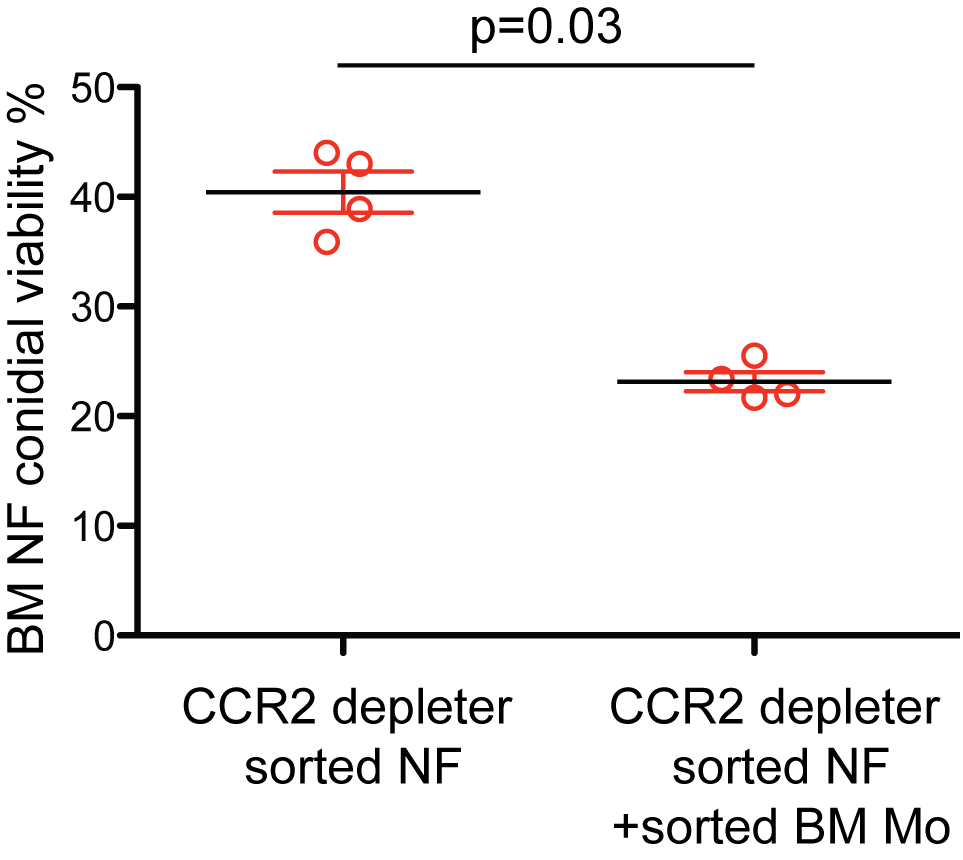

Supplement: Figure S1 — Killing of neutrophils isolated from CCR2 depleter mice is restored by culture with monocytes in vitro. Neutrophils were FACS sorted from the bone marrow of CCR2 depleter mice treated with DT and cultured alone or together with sorted monocytes. Monocytes were FACS sorted from the bone marrow of CCR2-GFP reporter mice and cultured with neutrophils at 1∶4 Mo∶NF ratio. FLARE conidia were added at 1∶4 conidia∶cell ratio. Scattered plots from an experiment show the average frequency (± SEM) of conidia viability within the neutrophil gate examined 24 hours after culture initiation. Statistical analysis was done by Mann-Whitney test. (TIF) [file ppat.1003940.s001.tif]

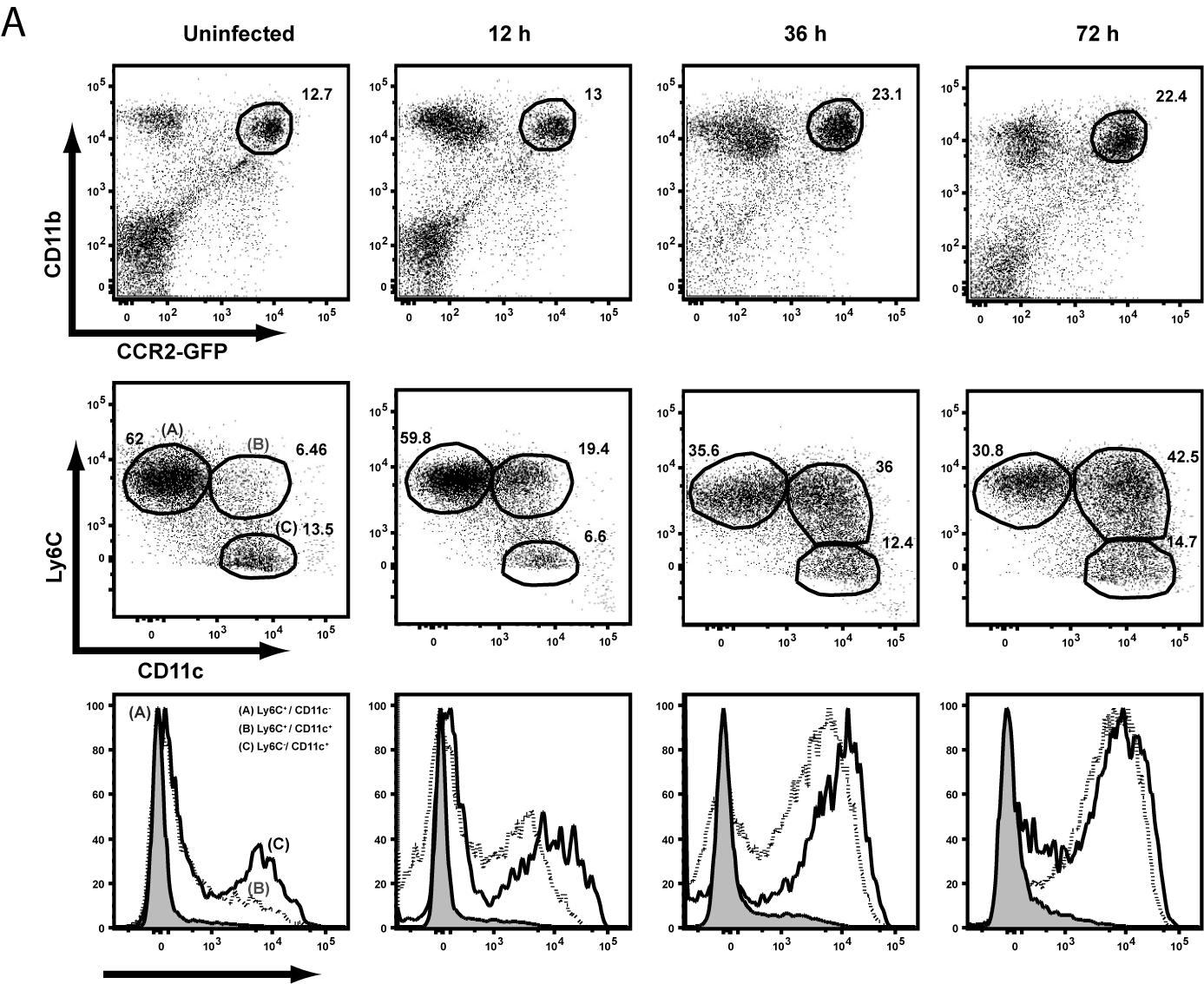

Supplement: Figure S2 — CCR2+Mo rapidly differentiate into Mo-DCs in response to A.fumigatus infection. CCR2-GFP reporter mice were infected with live A.fumigatus conidia and cell recruitment to the lung was examined at the indicated times. FACS plots are for one representative mouse. Top row: plots were gated on CD45+ cells, middle row: plots are gated on gates shown on top row, bottom row: MHC class II expression in populations A, B and C as gated on middle row panels. Data is representative of two independent experiments. (TIF) [file ppat.1003940.s002.tif]

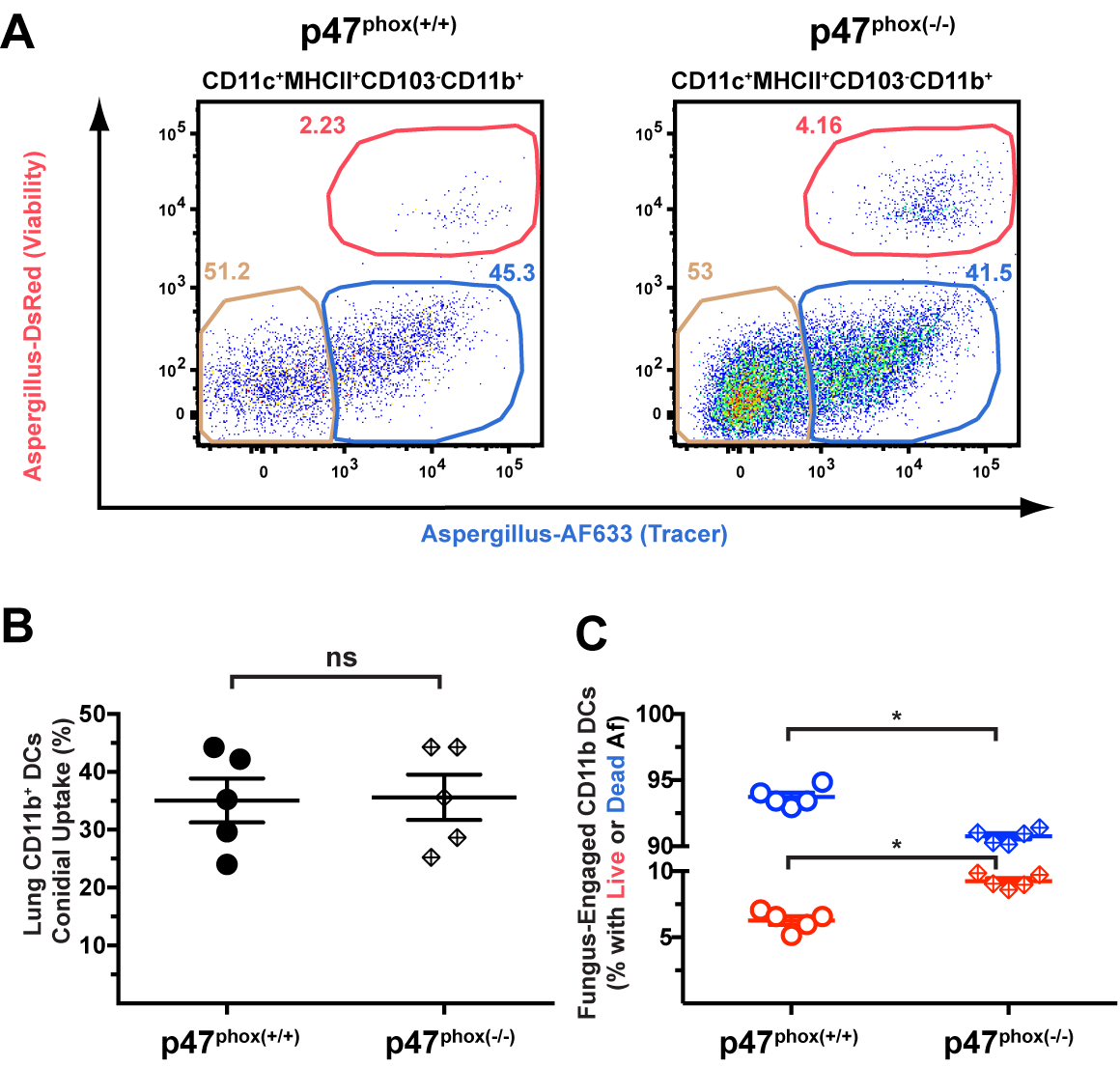

Supplement: Figure S3 — NADPH Oxidase mediates Mo-DC-dependent conidial killing in the lung. BM chimeric (1∶1 mix of CD45.1+ p47phox(+/+) and CD45.2+ p47phox(−/−) BM cells into irradiated CD45.1+CD45.2+ recipients) were infected with 3×10^7^ FLARE conidia. (A) Representative plots of p47phox(+/+) and p47phox(−/−) CD11b DCs (CD45+MHCII+CD11c+CD103−CD11b+) analyzed for dsRed and AF633 fluorescence show the frequencies of CD11b DCs that contain live (red gate) or killed (blue gate) conidia 36 h p.i. (B and C) Scattered plots from an experiment show the average frequency (± SEM) of CD11b DC (B) conidial uptake (R1+R2) and (C) conidial viability (R1/(R1+R2) in p47phox(+/+) and p47phox(−/−) cells. *p<0.05 by paired t-test. (TIF) [file ppat.1003940.s003.tif]
